# Gastrointestinal stromal tumor with nephrotic syndrome as a paraneoplastic syndrome: a case report

**DOI:** 10.1186/1752-1947-8-108

**Published:** 2014-03-27

**Authors:** Kiyoko Takane, Yutaka Midorikawa, Shintaro Yamazaki, Takahiro Kajiwara, Naoki Yoshida, Yoshiaki Kusumi, Tadatoshi Takayama

**Affiliations:** 1 Department of Digestive Surgery, Nihon University School of Medicine, 30-1 Oyaguchi Kami-machi, Itabashi-ku, Tokyo 173-8610, Japan; 2 Department of Pathology, Nihon University School of Medicine, 30-1 Oyaguchikami-machi, Itabashi-ku, Tokyo 173-8610, Japan

**Keywords:** Gastrointestinal stromal tumor, Nephrotic syndrome, Paraneoplastic syndrome

## Abstract

**Introduction:**

Paraneoplastic syndromes are disorders associated with clinical signs and symptoms caused by substances produced by malignant disease and are not directly related to the physical effects of a primary or metastatic tumor. We describe a patient with gastrointestinal stromal tumor of the stomach accompanied by nephrotic syndrome as paraneoplastic syndrome in whom symptomatic treatment was ineffective. Nephrotic syndrome caused by gastrointestinal stromal tumors is quite rare, and to the best of our knowledge this is the first time that such a case has been documented.

**Case presentation:**

We describe a 69-year-old Asian woman with a gastrointestinal stromal tumor of the stomach accompanied by paraneoplastic syndrome. The patient had severe hypoalbuminemia and proteinuria, which were apparently attributed to a gastrointestinal stromal tumor. After preoperative treatment for hypoalbuminemia, the tumor was resected and nephrotic syndrome improved. Two years after her operation, she is still alive with neither tumor recurrence nor nephrotic syndrome.

**Conclusion:**

Patients with refractory nephrotic syndrome caused by a malignant tumor should be treated aggressively, even if they are in poor general condition. Otherwise, the opportunity for potentially curative surgery may be missed.

## Introduction

Paraneoplastic syndrome (PNS) is a disorder or symptom caused by cancer or a reaction to a tumor, but is not due to the local presence of cancer cells [1,2]. PNS can resolve after complete tumor resection or a response to chemotherapy and recur in association with tumor recurrence. Clinical manifestations are mediated by hormones or cytokines excreted by tumor cells or by an immune response against the tumor [3].

The signs and symptoms of PNS are diverse, but common features include neuropathy, skin disease, and nephrotic syndrome. Peripheral neuropathy is one of the most frequent types of PNS of the nervous system and is usually associated with slight loss of muscle strength, hypoesthesia, and areflexia [4]. Itching paresthesia is the most common cutaneous symptom, and such symptoms may improve after treatment of the primary disease [1]. PNS is common among middle-aged and elderly patients who harbor cancers of the lung, breast, or ovaries, or lymphoma as the primary disease [5].

We describe a patient with gastrointestinal stromal tumor (GIST) of the stomach accompanied by nephrotic syndrome as PNS in whom symptomatic treatment was ineffective, but clinical remission was obtained after an operation. To the best of our knowledge, this is the first time such a case has been documented. We also discuss the incidence, diagnosis, and treatment of GIST accompanied by PNS.

## Case presentation

**Figure 1:**
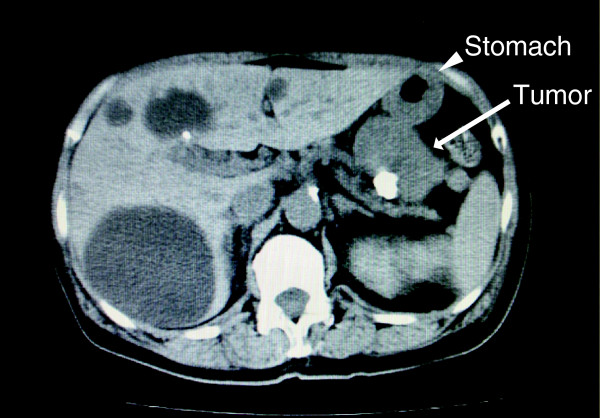
**Computed tomographic findings of the tumor.** A computed tomographic scan showing a large tumor measuring 56 × 55mm accompanied by calcification. An arrow indicates the tumor; the arrow head indicates the stomach.

**Figure 2:**
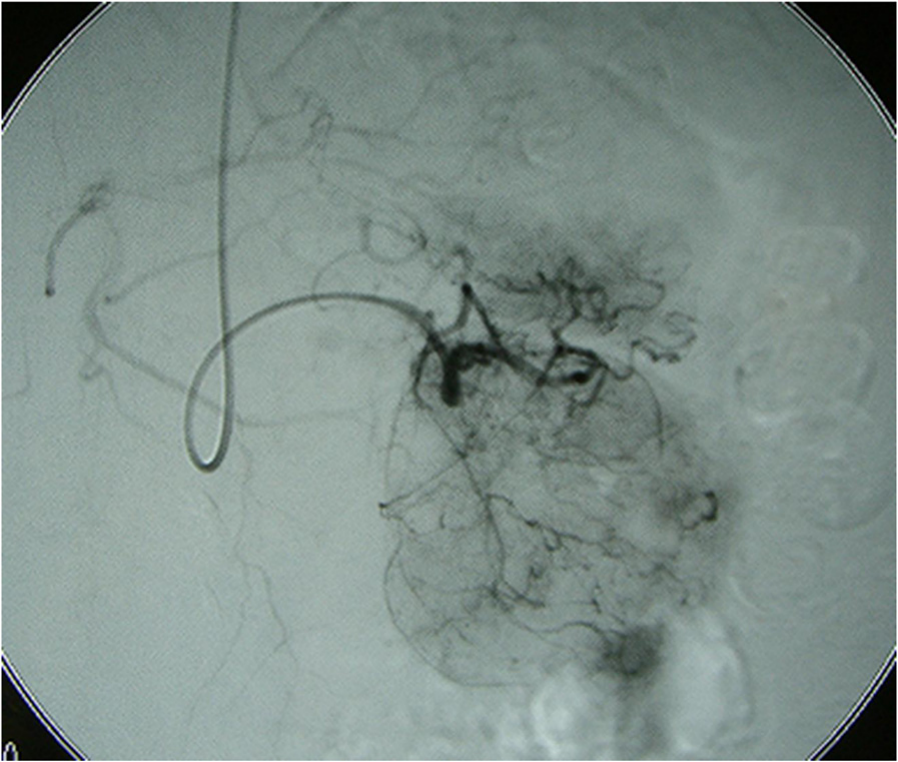
**Angiographic findings of the tumor.** Angiography via the left gastric artery showed staining of the tumor.

**Figure 3:**
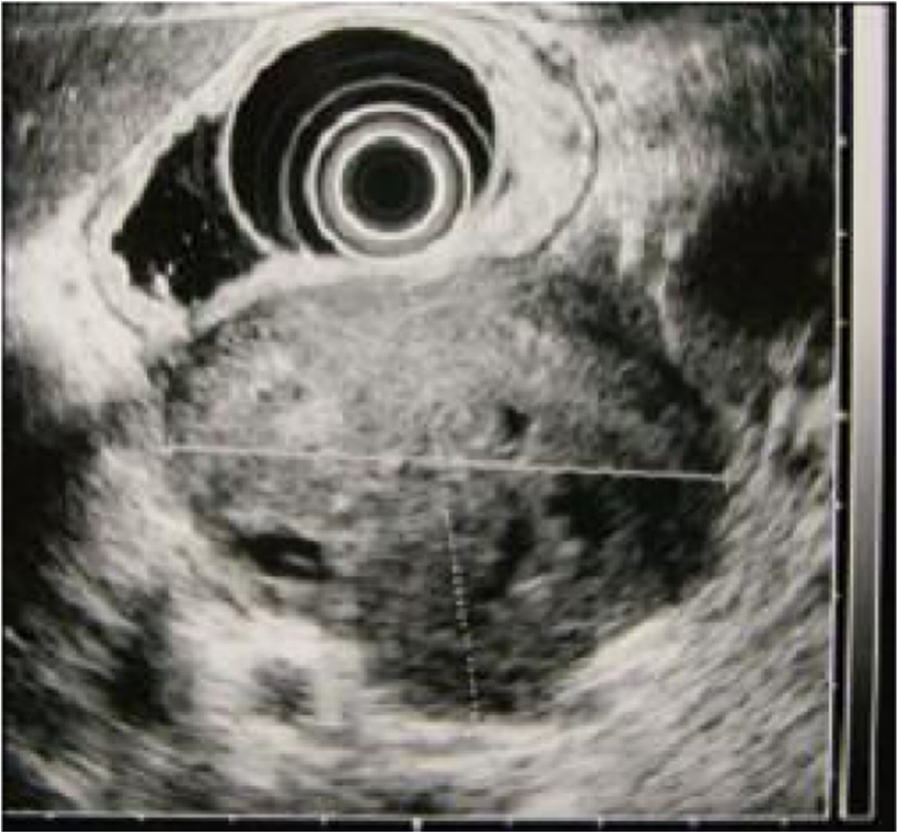
**Endoscopic ultrasonographic findings of the tumor.** Endoscopic ultrasonography demonstrated a heterogeneous lesion 60mm in diameter, arising in the posterior wall of the stomach.

A 69-year-old Asian woman was admitted to our hospital because of diminished appetite. She had no previous medical history and was not complaining of fever and, therefore, she was not taking oral non-steroid anti-inflammatory drugs. Computed tomography revealed a gastric tumor measuring 56 × 55mm, accompanied by calcification (Figure 1). Angiography via her left gastric artery showed staining of the tumor (Figure 2), and endoscopic ultrasonography demonstrated a heterogeneous lesion 60mm in diameter, arising in the posterior wall of the stomach (Figure 3). Laboratory examinations revealed hypoproteinemia (4.8g/dL; normal range, 6.5 to 8.0), hypoalbuminemia (2.2g/dL; normal range, 3.8 to 5.3), and severe proteinuria (8.47g/dL; normal range, 0 to 0.15), and her protein creatinine ratio was 14.3g/g and amount of urine for 24 hours was 1200mL, clinically suggesting nephrotic syndrome. Therefore, to avoid an invasive inspection, a kidney biopsy was not carried out. There were no neurologic symptoms or skin disease.

**Figure 4:**
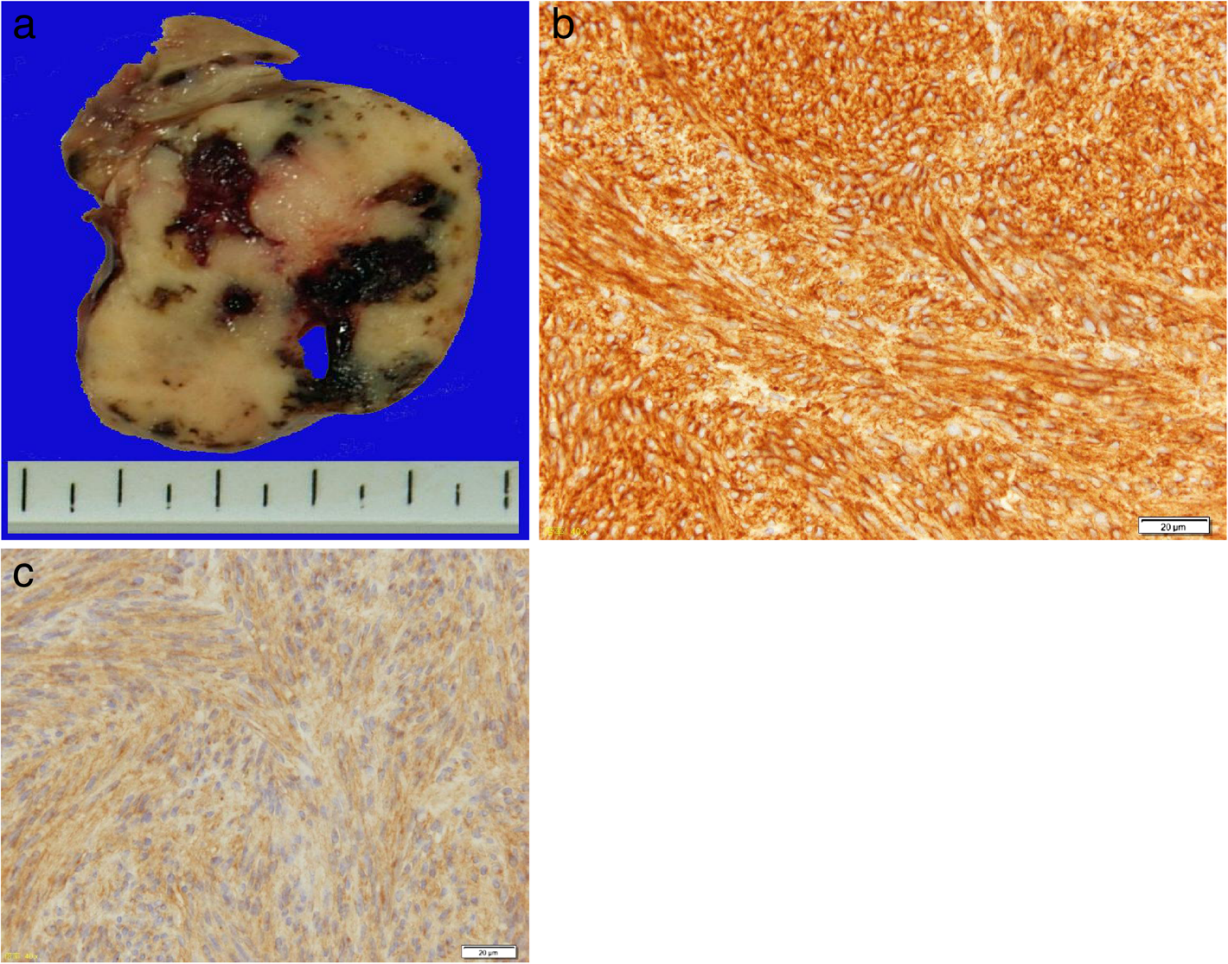
**Pathological findings of the tumor. (a)** Macroscopically, the tumor appeared to be gray-whitish and hard and arose from the gastric wall. **(b,c)** Both CD34 and c-kit were positive on immunohistochemical staining.

Despite albumin administration, her hypoalbuminemia did not improve preoperatively. She was given a diagnosis of GIST of the stomach accompanied by nephrotic syndrome, and partial gastrectomy was performed. Macroscopically, a well-demarcated, hard, whitish submucosal tumor arose from the gastric wall (Figure 4a); histological and immunohistochemical examinations revealed a spindle-cell tumor positive for CD34 and c-kit (Figure 4b, 4c), and a GIST was diagnosed.

Her postoperative course was uneventful. One week after her operation, her serum albumin (3.3g/dL) and urinary protein (1.71g/dL) levels remarkably improved after removal of the tumor. She remains well with no recurrence of the tumor or nephrotic syndrome (her recent urinary protein level: 1.19mg/mL) 2 years after surgery.

## Discussion

Nephrotic syndrome can occur as malignancy-associated PNS, and previous studies have estimated that cancer occurs in 11% to 22% of patients with nephrotic syndrome [2,6,7]. Therefore, if an adult presents with nephrotic syndrome, physicians should bear in mind the risk of underlying malignant disease. Gastric cancer, lung cancer, and malignant lymphoma are frequently associated with nephrotic syndrome of which the rates are 25, 15 and 10%, respectively [8]. To the best of our knowledge, GIST has not been previously reported as an underlying disease of PNS, and this is the first such case to be documented.

In patients with PNS, surgical tumor removal can be followed by the regression of nephrotic syndrome. One study reported that nephrotic syndrome improved after operation in 78% of patients [4]. Therefore, surgical resection should be performed even in the presence of poor general condition due to PNS, because cancer is a potential cause of nephrotic syndrome [9]. The hypoalbuminemia and proteinuria in our patient did not preoperatively respond to symptomatic treatment. By contrast, clinical remission of nephrotic syndrome was obtained immediately after tumor removal. Considering that the patient did not take drugs perioperatively which cause nephrotic syndrome, such as non-steroidal anti-inflammatory drugs or tyrosine kinase inhibitors, the nephrotic syndrome was PNS associated with a GIST of the stomach.

Hypothetical explanations have been proposed to link minimal-change glomerulopathy to solid tumors. One potential mechanism involves the secretion of some tumoral factor that increases glomerular permeability without affecting glomerular capillary structure, resulting in massive proteinuria. Alternatively, tumor-specific antigens such as carcinoembryonic antigens may react with antibodies to form immune complexes, which accumulate as deposits in the glomerular basement membrane and cause renal dysfunction. The mechanism of this phenomenon can involve type III allergy [10].

In summary, we have documented a case of GIST of the stomach that was associated with nephrotic syndrome as PNS. Patients with refractory nephrotic syndrome caused by a malignant tumor should be treated aggressively to improve signs and symptoms associated with PNS. Otherwise, the opportunity for potentially curative surgery may be lost.

## Conclusions

Nephrotic syndrome caused by GISTs is quite rare, and to the best of our knowledge this is the first time that such a case has been documented. Patients with refractory nephrotic syndrome caused by a malignant tumor should be treated aggressively, even if they are in poor general condition. Otherwise, the opportunity for potentially curative surgery may be missed.

## Consent

Written informed consent was obtained from the patient for publication of this case report and any accompanying images. A copy of the written consent is available for review by the Editor-in-Chief of this journal.

## Abbreviations

GIST: Gastrointestinal stromal tumor; PNS: Paraneoplastic syndrome.

## Competing interests

The authors declare that they have no competing interests.

## Authors’ contributions

YM was a major contributor in writing the manuscript. TK, NY, SY and TT performed the surgical procedure and obtained the patient data. YK performed the pathologic examination and was a contributor in writing the manuscript. All authors read and approved the final manuscript.
